# Carbon Dioxide Pressure and Catalyst Quantity Dependencies in Artificial Photosynthesis of Hydrocarbon Chains on Nanostructured Co/CoO Surfaces

**DOI:** 10.3390/molecules29071481

**Published:** 2024-03-27

**Authors:** Zhe Kan, Zibo Wang, Haizhou Ren, Mengyan Shen

**Affiliations:** 1 Department of Physics and Applied Physics, University of Massachusetts Lowell, One University Avenue, Lowell, MA 01854, USA; 2 Center for Advanced Manufacturing of Polymers and Soft Materials, University of Massachusetts Lowell, One University Avenue, Lowell, MA 01854, USA

**Keywords:** photosynthesis, nanostructure, hydrocarbons, carbon dioxide

## Abstract

In this study, we investigated the influence of pressure and the quantity of Co/CoO catalyst on an artificial photosynthesis process that converts CO_2_ and H_2_O into hydrocarbons (C*_n_*H_2*n*+2_, where *n* ≤ 18). The adsorption of CO_2_ and H_2_O on Co/CoO surfaces proved to be pivotal in this photo-catalytic reaction. Photoexcited carbon dioxide and water molecules ((CO_2_)* and (H_2_O)*) generated by illuminating the catalyst surface led to the formation of alkene hydrocarbon molecules with carbon numbers following an approximate Poisson distribution. The optimal pressure was found to be 0.40 MPa. Pressure less than 0.40 MPa resulted in low CO_2_ adsorption, impeding excitation for photosynthesis. At greater pressure, oil/wax accumulation on Co/CoO surfaces hindered CO_2_ adsorption, limiting further photosynthesis reactions. The average number of carbon atoms in the hydrocarbons and hydrocarbon yield were correlated. The amount of Co/CoO was also found to affect the hydrocarbon yield. Our study contributes to the understanding of Co/CoO-catalyzed photosynthesis and suggests that an open-flow system could potentially enhance the productivity of long-chain hydrocarbons.

## 1. Introduction

The synthesis of clean, sustainable fuel is an important topic. A well-known example is the production of hydrogen [1,2,3,4,5,6,7,8,9] which can be achieved through the splitting of water molecules. A different process for synthesizing fuel is artificial photosynthesis. Artificial photosynthesis emulates processes observed in nature where plants harness sunlight, carbon dioxide, and water to make energy-rich compounds. In natural photosynthesis, sunlight converts water and carbon dioxide into glucose—a vital source of energy for plants. With artificial photosynthesis, researchers seek to engineer systems that can capture solar energy to create high-energy chemical fuels. Artificial photosynthesis is a carbon-neutral approach that consumes carbon dioxide while curbing detrimental greenhouse gas emissions. A noteworthy example is the production of methanol [10,11,12,13,14]. To advance the field, research has focused on nanostructure-based methodologies for enhancing hydrocarbon generation [15,16,17,18,19,20,21]. However, challenges persist, and new innovations are needed for energy-efficient catalytic systems.

Various long-chain hydrocarbons have been successfully synthesized using Co/CoO nanostructures in the presence of water, carbon dioxide, and solar irradiation [22,23,24]. Experiments involving hydrogen and carbon isotopes have been undertaken to track the formation of hydrocarbon products [22,23]. However, understanding the precise formation mechanism of these hydrocarbons remains a challenge [25,26,27] impeding the optimization that is essential for practical applications.

Temperature, pressure, and catalyst amount are crucial factors for the artificial photosynthesis process. In our previous study, we have explored the temperature dependence of the process with the nanostructured Co/CoO catalyst and found that carbon dioxide (CO_2_) and water (H_2_O) transform into hydrocarbons within a temperature range of 58 to 242 °C [24]. Experimental findings indicate that alkane hydrocarbons with the formula C_n_H_2n+2_ and 3 ≤ n ≤ 16 predominantly emerge at temperatures exceeding 60 °C. Those molecules are significantly larger than ones in references where photothermal effects play important roles [25,26]. Maximum productivity is observed at about 130 °C followed by a sharp decline beyond this point. Production gradually increases up to 220 °C, after which benzene (C_6_H_6_) and its derivatives are formed, including toluene (C_7_H_8_), p-xylene (C_8_H_10_), and C_9_H_12_.

Analysis of the temperature-dependent reaction rate model reveals that the vaporization of adsorbed water contributes to the observed production peak. The activation energy is estimated to be approximately 1 eV, aligning with the carbon monoxide (CO) and H_2_ reaction to synthesize chain hydrocarbons [24,28,29,30,31,32]. The experimental results suggest chemisorbed CO_2_ and physisorbed H_2_O on the Co/CoO surface undergo dissociation or excitation upon exposure to light. The disassociated or excited molecules then react to form hydrocarbons. As the temperature exceeds 220 °C and most water molecules leave the Co/CoO surface, the hydrogen source becomes significantly diluted, while the carbon source remains consistent due to chemisorption. Consequently, benzene and its derivatives with a lower hydrogen atom ratio are formed.

With fixed temperature and sufficient water molecules, we explore the pressure dependence of CO_2_ in artificial photosynthesis. This study examines the interplay between carbon dioxide pressure, catalysis amounts, and their impact on the process. We show that hydrocarbon chain synthesis occurs specifically on the surfaces of Co/CoO.

## 2. Results and Discussion

**Figure 1:**
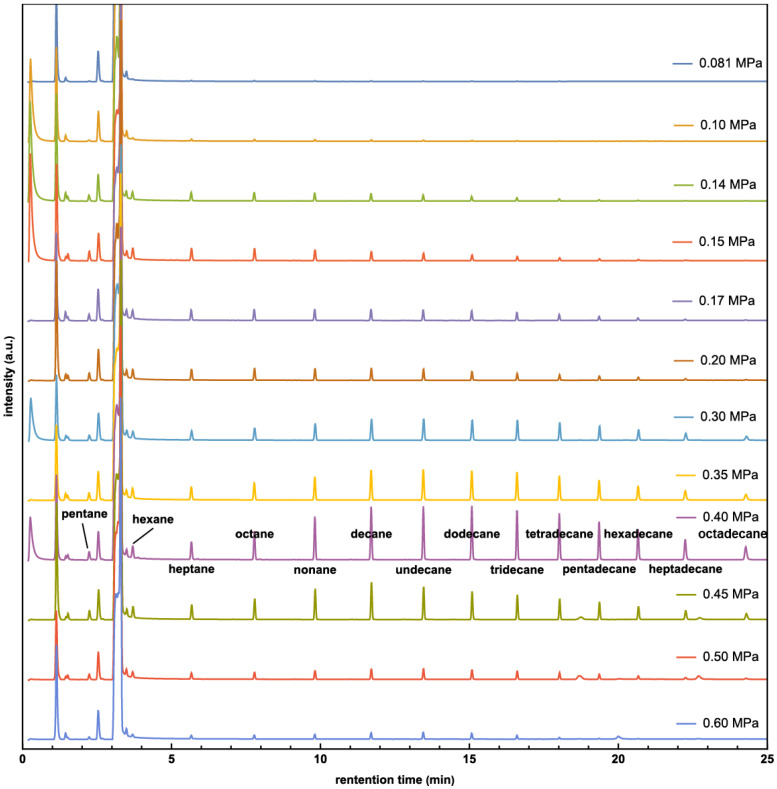
GC spectra of products with pressure from 0.081 MPa to 0.60 MPa.

**Figure 2:**
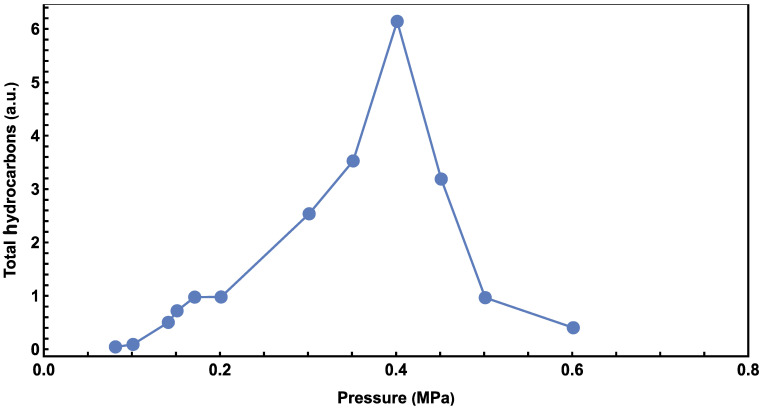
Relationship between total alkene hydrocarbons and CO_2_ pressure.

Gas chromatograph (GC) results at different pressures are presented in Figure 1. Invalid results (e.g., caused by leaked vessels) have been removed. Hydrocarbons ranging from pentane to octadecane appear near 0.45 MPa. Experiments at 0.40 MPa were found to have the most products, and a comprehensive mass spectroscopy (MS) analysis of the peaks confirmed the presence of hydrocarbons, from pentane to octadecane, in every experiment (please see the Supporting Materials). The intensities of hydrocarbons in the experiments were not calibrated, so arbitrary units (a.u.) are used for intensity comparison. The total hydrocarbon amount at various pressures is shown in Figure 2, from which the highest hydrocarbons production is also found at 0.40 MPa. Below this pressure, the production of hydrocarbons increases with CO_2_ pressure but decreases when the pressure exceeds 0.4 MPa. Similar trends for CO_2_ pressure dependence have been reported previously [33].

**Figure 3:**
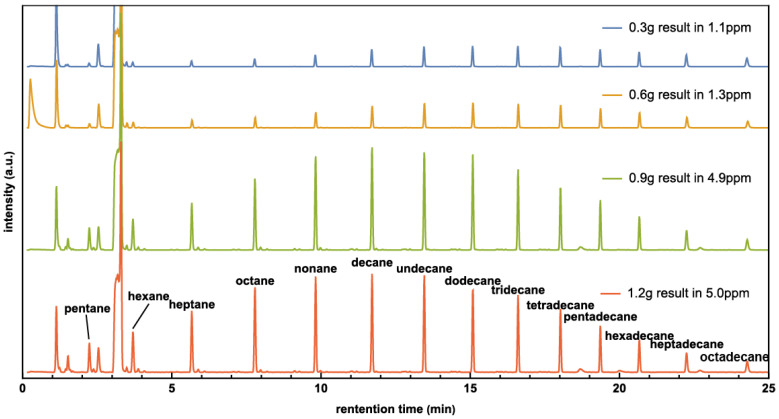
The GC of hydrocarbon products carried with Co/CoO amount from 0.3 g to 1.2 g.

In another set of experiments, the amount of Co/CoO catalyst was varied while keeping other parameters constant. A pressure of 0.40 MPa was chosen, as it yielded the highest productivity in previous experiments. The results were analyzed using GC-MS, showing that the product strongly depends on catalyst amount, where the highest quantity of hydrocarbons can be produced around 0.9 g and 1.2 g Co catalysts, as shown in Figure 3. The result suggests that the reaction has been adequately catalyzed, and additional catalyst may not significantly enhance productivity. This occurs because the catalyst accumulates in layers. Catalyst particles in lower layers do not receive as much light, which impedes their participation in the artificial photosynthesis process. 

Experiments also show that the peak of alkene hydrocarbons shifts to shorter molecules as the catalyst amount increases. For example, the peak value in the 0.3 g experiment was around tridecane (C_13_H_28_), while the peak value in the 1.2 g experiment was around decane (C_10_H_22_). 

The adsorbed CO_2_ molecules can be excited or dissociated by obtaining photoelectrons around the conduction band of CoO, while a surface-plasmon effect around nano-Co structures enhances local excitation [22]. This excitation results in the formation of long chain hydrocarbons from excited carbon dioxide molecules (CO_2_)* and the excited water molecules (H_2_O)* on the Co/CoO surface:

(1) $${CO}_{2}+H_{2}O\overset{photo-excited\;catalyst}{\to }\left({CO}_{2}\right)*+\left(H_{2}O\right)*\to C_{n}H_{2n+2}$$

where n is the number of carbon atoms, ranging from 5 to 17 in our GC data. The quantity of Co/CoO catalyst plays a crucial role in the adsorption dynamics of CO_2_ molecules on the surface. A lower catalyst amount results in a higher density of adsorbed CO_2_ molecules, leading to a higher concentration of produced (CO_2_)*, which favors the formation of long hydrocarbon molecules and vice versa. Assuming molecules formed have *u* carbon atoms on average, and hydrocarbons with n carbon atoms were formed in a certain interval, a Poisson distribution may effectively describe this process:

(2) $$P\left(n\right)=\frac{u^{n}}{n!}Exp\left(-u\right)$$

**Figure 4:**
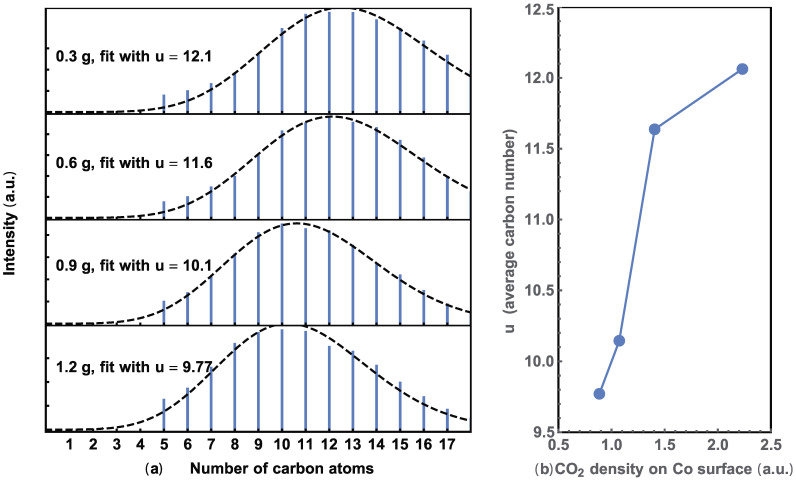
(**a**) Extracted alkene hydrocarbons with different amounts of Co/CoO and their Poisson fit. (**b**) Relationship between the average number of u and CO_2_ density on the surface area of the catalyst.

(3) I(n)=P(n)(14n+2)

where P(*n*) is the probability of finding C*_n_*H_2*n*+2_ in the result alkane hydrocarbons, this distribution depends on the occurrence of (CO_2_)* formation on the catalyst. As the signal intensities of the GC and MS are both proportional to the mass of the molecules, the GC spectra is fitted with I(n) in Equation (3) (please read the Support Materials), as shown in Figure 4. Here, the factor (14*n* + 2) is the mass of C*_n_*H_2*n*+2_. 

The number of carbon atoms vs. normalized density was calculated from the GC data. As shown in Figure 4a, the dashed line represents the best fit using Equation (3) for each alkane hydrocarbon distribution. The peak position shifts to a lower n value with increasing catalyst. These trends indicate a decrease in u as carbon atoms are shared by increasing the quantity of catalysts. This trend is more obvious when the average u is plotted against the CO_2_ concentration on the surface of the catalyst, as shown in Figure 4b. The total surface area of the Co/CoO catalyst was calculated from its weight, assuming the particles are uniform spheres that adsorb CO_2_ uniformly. 

**Figure 5:**
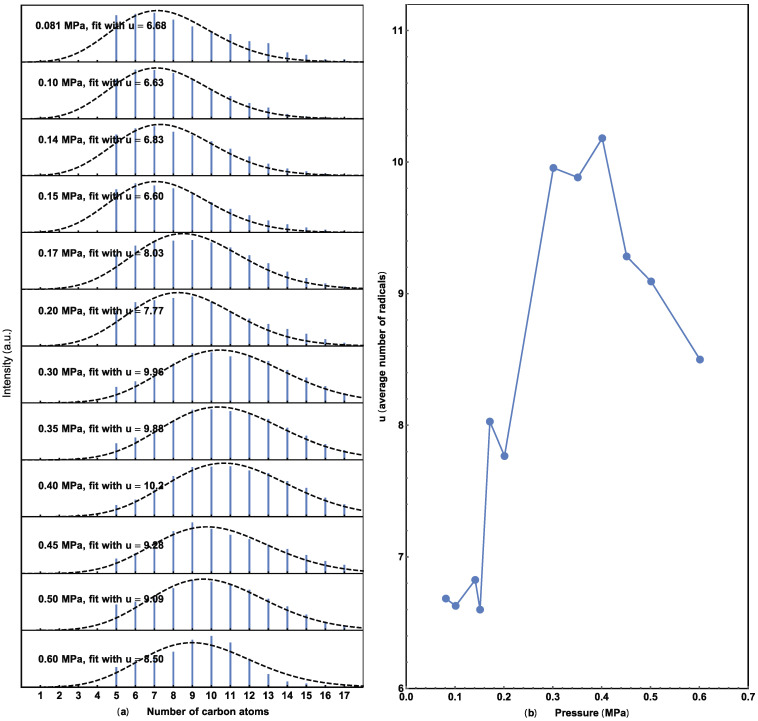
(**a**) Extracted alkene hydrocarbons with different CO_2_ pressures and their Poisson fit. (**b**) Relationship between the average number of u and the pressure. Average carbon number versus CO_2_ pressure from 0.08 to 0.60 MPa.

Figure 5 provides a similar Poisson analysis of the pressure data in Figure 1, to better illustrate the relationship between the average carbon number and CO_2_ pressure. The carbon number peaks at 0.40 MPa, increases as the CO_2_ pressure increases below this value, and decreases afterward, which is similar to the results shown in Figure 2. This similarity agrees with the concept that higher adsorbed CO_2_ concentrations result in higher production and a longer average hydrocarbon molecule.

Adsorbed CO_2_ and H_2_O on the Co/CoO surfaces play an important role [3]. The reaction rate of adsorbed CO_2_ and H_2_O molecules may also follow the form of a power law [34]:

(4) $$r=\kappa {\left[{CO}_{2}\right]}^{m}{\left[H_{2}O\right]}^{l}$$

where the square bracket represents the concentration of CO_2_ or H_2_O. The exponents *m* and *l* are the partial orders of reaction and may be fractional. κ is the rate constant, which has a temperature dependence and is usually described by an Arrhenius equation characterized by an activation energy [35], approximately 1 eV in our previous research [24]. In this study, the reaction was conducted under an isotherm process. The adsorbed [CO_2_] generally depends on the pressure of CO_2_, and the Freundlich adsorption isotherm serves as a good approximation [36,37,38]: adsorbed [CO_2_] is proportional to *p^x^*, where p is pressure of CO_2_ and *x* depends on temperature and surface details. Given a constant H_2_O partial pressure, [H_2_O] is consistent in this experiment. Therefore, the reaction rate can be expressed as follows:

(5) $$r(p)=Ap^{B}$$

where *A* is an arbitrary coefficient, B = mx is a power factor to represent the pressure effects. As shown in Figure 2, when the CO_2_ pressure is lower than 0.40 MPa, the hydrocarbon production increases as the pressure increases, and Equation (5) can be used to fit and analyze the data. 

**Figure 6:**
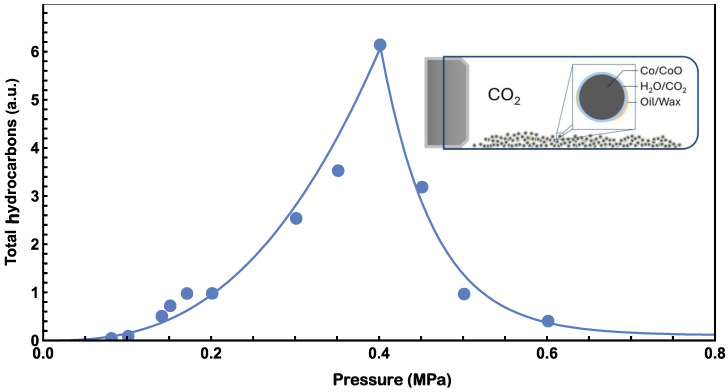
Best fit of pressure data from Figure 2 with Equation (5) for pressure lower than 0.4 MPa and Equation (6) for pressure higher than 0.4 MPa. The inset diagram illustrates the model’s behavior when the pressure in the reaction chamber exceeds 0.4 MPa. At higher pressures, oil/wax hydrocarbons may accumulate on the Co/CoO surfaces, leading to a reduction in the reaction rate for artificial photosynthesis.

The increase in the production of hydrocarbon products at low pressure levels can be understood using the power law described above. However, at pressures higher than 0.4 MPa, the product yield decreases with increasing pressure. This abnormal decrease in production may be explained by the physical properties of hydrocarbons. At our reaction temperature, hexane exhibits a vapor pressure of approximately 0.45 MPa estimated with Antoine’s equation [39]. Our objective is to identify hydrocarbons with higher molecular weight and lower vapor pressure than hexane. Beyond 0.45 MPa, most produced hydrocarbons exist in oil/wax form [40], hindering CO_2_ adsorption on Co/CoO catalyst surfaces as shown in the inset of Figure 6. Assuming an average vapor pressure (*p*_c_) of 0.40 MPa for potential product hydrocarbons, their accumulation on the Co/CoO surface decreases the reaction rate with increasing pressure. To analyze experimental data at pressures exceeding 0.40 MPa, the following empirical function may be used to estimate the reaction rate:

(6) $$r\left(p\right)=M*exp\left(-\frac{C(p-p_{c})}{p_{c}}\right)+D$$

where *M* is the reaction rate at 0.40 MPa, *C* is a pressure power factor greater than 1, and *D* is the minimum reaction rate in high-pressure experiments. *D* also indicates the amount of oil/wax hydrocarbon sufficient to prevent CO_2_ from dissolving in water during this series of experiments. Our best fits for low- and high-pressures are plotted in Figure 6. 

We found that the following parameters, *A* = 70, *B* = 2.7, *C* = 6.3, *D* = 0.11, fit the data well in low- and high-pressure ranges, as plotted in the solid line of Figure 6. In the low-pressure range, *A* is an arbitrary unit parameter for the reaction rate, and *B* represents the enhanced effect from pressure. Since CO_2_ concentration is directly proportional to pressure, a *B* value greater than one, 2.7 in this case, indicates that there could be other pressure-enhancing effects. In the high-pressure range, *M* was determined by ensuring consistency with the low-pressure data, whilst maintaining the continuity of the fitting curve with Equations (5) and (6) at the *p_c_*, 0.4 MPa. *C* represents the hindering effect from oil/wax, and it is found to be significantly higher than 1. *D* represents the minimum amount of hydrocarbon generated from high pressure experiments. The data in Figure 1 indicated zero hydrocarbon generation when the pressure approaches zero. However, it is anticipated that there will be a non-zero limit in the high-pressure range. This expectation arises from the influence of the *D* term in Equation (6). The accumulation of oil/wax hydrocarbons on the surfaces of Co/CoO also blocks the photosynthesis of hydrocarbons from CO_2_ and H_2_O and contributes to the decreasing trend. 

The results in Figure 3 show a correlation between higher production and a smaller average carbon number, seemingly contradicting the concept derived from Figure 2 and Figure 4, which proposes that a higher concentration of adsorbed CO_2_ leads to increased production and a larger average hydrocarbon molecule. However, the experiment in Figure 3 demonstrates that as the amount of Co/CoO increases, production also increases. In the above discussion, we simplify the argument by stating that under the same CO_2_ pressure condition, there is a higher density of adsorbed CO_2_ for a smaller amount of Co/CoO. Nevertheless, since the CO_2_ pressure is kept at 0.4 MPa, where there are sufficient CO_2_ molecules for adsorption, the density of the adsorbed CO_2_ should not change significantly. Additionally, the effective light radiation areas were comparable. As a result, the average radiation intensity onto the catalyst surface decreases as the amount of Co/CoO increases. Therefore, “a higher concentration of excited (CO_2_)* adsorbed on Co/CoO leads to increased hydrocarbon production and a larger average hydrocarbon molecule.” This statement succinctly encapsulates the experimental findings. 

The CO_2_ pressure dependence with varying catalyst quantities will provide additional insights into the optimal pressure. Further detailed investigation is required to fully understand this relationship, which is expected to resemble that shown in Figure 6, allowing us to determine both the optimal pressure and quantity.

## 3. Materials and Methods

The materials are the same as those described in our previous work [22,23,24]. The Co powder, obtained from Goodfellow, consisted of micro-particles with diameters ranging from 50 to 100 μm. These particles were mixed with a 3.5% aqueous solution of hydrogen chloride (HCl) for 10 min, followed by rinsing with distilled water. Acid etching removed the oxidation layers on the Co particle surfaces, and the resulting catalyst was degassed under vacuum for about 1 h to eliminate any adsorbed carbon dioxide on the surfaces. Subsequently, the Co powder was exposed to air at room temperature for 2 h, leading to the formation of nanoflakes on the particle surfaces. 

The methods are also the same as those described in our previous work [22,23,24] For the experiments at different CO_2_ pressures, two grams of catalyst were loaded into a 20 mL glass reactor, and 350 mg of distilled water was added to cover the catalyst. The reactor was then evacuated to a pressure of approximately 1 × 10^−3^ atm and filled with CO_2_ to various absolute pressures, ranging from 0.08 MPa to 0.6 MPa, where 0.081 MPa represents a degassed vessel. The pressures were measured at a temperature of 20 °C. For experiments involving varying amounts of catalyst (ranging from 0.3 to 1.2 g), a CO_2_ pressure of 0.4 MPa was selected according to the result of the pressure dependence experiments. 

The glass reactor was sealed throughout the reaction. It was positioned horizontally and manually shaken, ensuring the even distribution of cobalt particles on the lower half of its cylindrical wall. A solar lamp (Solar Simulator SOL 500, Honle, Nicolaus-Otto-Str. 2, D-82205 Gilching, Germany), designed to simulate natural sunlight, was utilized on the nanostructured Co microparticles within the sealed reactor. During irradiation, the reactor was placed on a stage tilted at an angle of 10° to the surface below the solar lamp. This angle is crucial to prevent water vapor from condensing near the cap, which would otherwise make the cobalt particles too dry to react if the reactor was laid horizontally. Tilting the reactor allowed condensed water to flow back to the cobalt particles, ensuring adequate moisture during the experiment. 

The solar lamp was situated about 15 cm away from the glass vial, providing a light intensity of 100 mW/cm^2^, mimicking sunlight intensity. To maintain a temperature significantly higher than room temperature, the bottom half of the cylindrical reactor was wrapped with materials such as fiberglass, paper, rubber, or an aluminum sheet. These materials were used to regulate the temperature to 120 °C for samples at different CO_2_ pressures, and thermal couples were used to monitor the temperature. To eliminate the potential influence of contamination, a control experiment was conducted using the same setup without light. After irradiating for 20 h, non-volatile organic products were extracted by injecting approximately 3 mL of dichloromethane (DCM) into the reactor. The extracted DCM was then analyzed using a Bruker Scion SQ gas chromatography-mass spectrometry (GC-MS) system equipped with a 30 m ZB-624 capillary column. In the gas chromatography spectra, hydrocarbons of varying molecular masses pass through a column with different retention times [41] as observed in Figure 1 and Figure 3. Generally, larger molecules exhibit longer retention times. The mass spectra of the hydrocarbon products have also been analyzed (please see the Supporting Materials).

## 4. Summary

We conducted experiments exploring the influence of pressure and Co/CoO amounts on an artificial photosynthesis reaction that converts CO_2_ and H_2_O into hydrocarbons. The adsorption of CO_2_ and H_2_O on Co/CoO surfaces is a critical factor. Photoexcited carbon dioxide and water molecules ((CO_2_)* and (H_2_O)*) on the catalyst surface result in alkane hydrocarbons with carbon numbers following an approximate Poisson distribution. Optimizing pressure in a closed vessel, we found that 0.40 MPa is the ideal pressure. Higher pressures led to oil/wax accumulation hindering CO_2_ adsorption, while lower pressures resulted in insufficient CO_2_ for excitation. A correlation between the average number of carbon atoms in hydrocarbons and hydrocarbon production was observed: higher production was associated with a greater number of carbon atoms. The Co/CoO amount dependence aligns with findings from pressure dependence experiments. However, adsorbed CO_2_ depends on pressure, total Co/CoO surface area, and the hindering effect of oil/wax. In the future, an open-flow system may be studied as a way to enhance productivity. More detailed investigations of CO_2_ pressure dependence for a range of catalyst amount, water quantity, and temperatures will offer valuable insight into the optimal conditions for artificial photosynthesis.

## Data Availability

Data is contained within the article or Supplementary Material.

## Supplementary Materials

The following supporting information can be downloaded at: https://www.mdpi.com/article/10.3390/molecules29071481/s1, Figure S1: Mass spectra of hydrocarbons ranging from pentane to octadecane of the products in Figure 1.
