# Supplementary material for: Carbon Dioxide Pressure and Catalyst Quantity Dependencies in Artificial Photosynthesis of Hydrocarbon Chains on Nanostructured Co/CoO Surfaces
Source: Molecules. 2024 Mar 27;29(7):1481. doi: 10.3390/molecules29071481 (PMC11013242; doi:10.3390/molecules29071481)
Supplement: Supplementary file 1 [file molecules-29-01481-s001.zip › molecules-2929839-supplementary.pdf]

Carbon Dioxide Pressure and Catalyst Quantity Dependencies in Artificial Photosynthesis of Hydrocarbon Chains on Nanostructured Co/CoO Surfaces

Zhe Kan [1], Zibo Wang [1], Haizhou Ren [1] and Mengyan Shen [1,2] *

1 Department of Physics and Applied Physics, University of Massachusetts Lowell, One University Avenue, Lowell Massachusetts 01854, United States
2 Center for Advanced Manufacturing of Polymers and Soft Materials, University of Massachusetts Lowell, One University Avenue, Lowell Massachusetts 01854, United States
* Correspondence: author: Mengyan_Shen@uml.edu

Support materials:

Figure s1 displays mass spectra of hydrocarbons ranging from pentane to octadecane, representing the products observed in our experimental setup, as illustrated in Figure 1 within this study. Each inset within the spectra showcases the mass of the entire molecule. The similarity of the mass distributions of molecular segments to standard data in the Bruker Scion SQ gas chromatography-mass spectrometry (GC-MS) system's library, along with their standard retention time, confirms the presence of linear hydrocarbons ranging from pentane to octadecanes. The system is designed so that the accumulated intensity of the bars in each mass spectrum is proportional to the intensity of its peaks in the GC spectra, enabling the utilization of Equation (3) for statistical analysis.

Figure s1 Mass spectra of hydrocarbons ranging from pentane to octadecane of the products in this work.

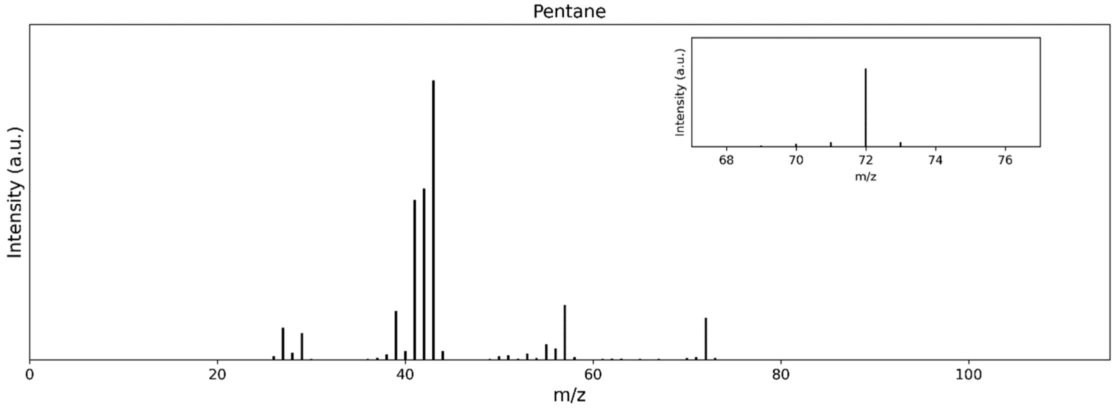

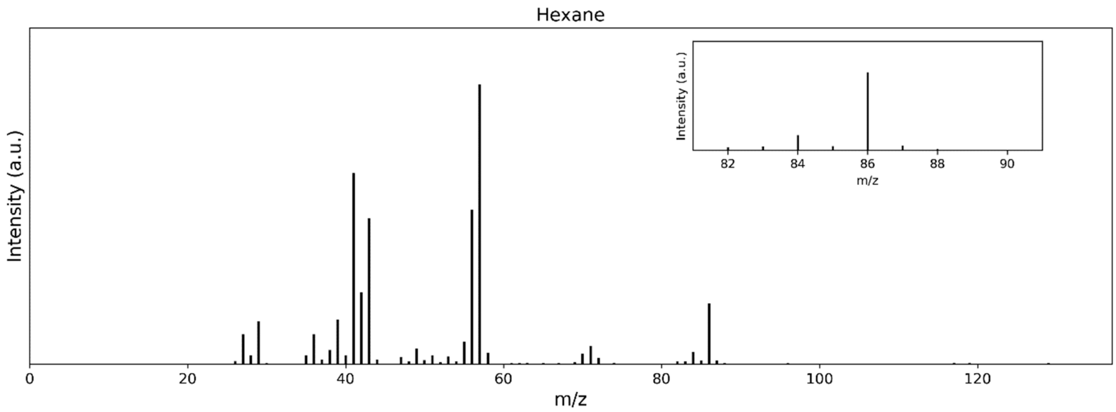
Hexane
Intensity (a.u.)
m/z
0
20
40
60
80
100
120
82
84
86
88
90

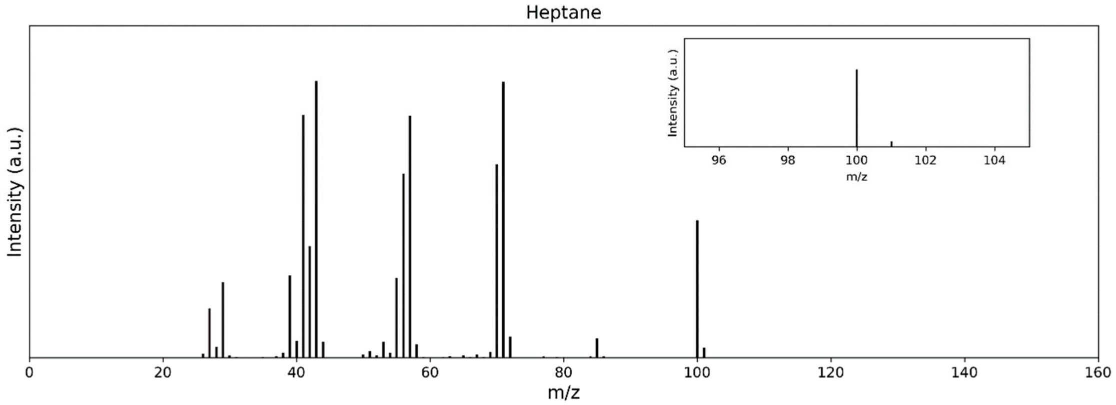
Heptane
Intensity (a.u.)
m/z
0
20
40
60
80
100
120
140
160
96
98
100
102
104

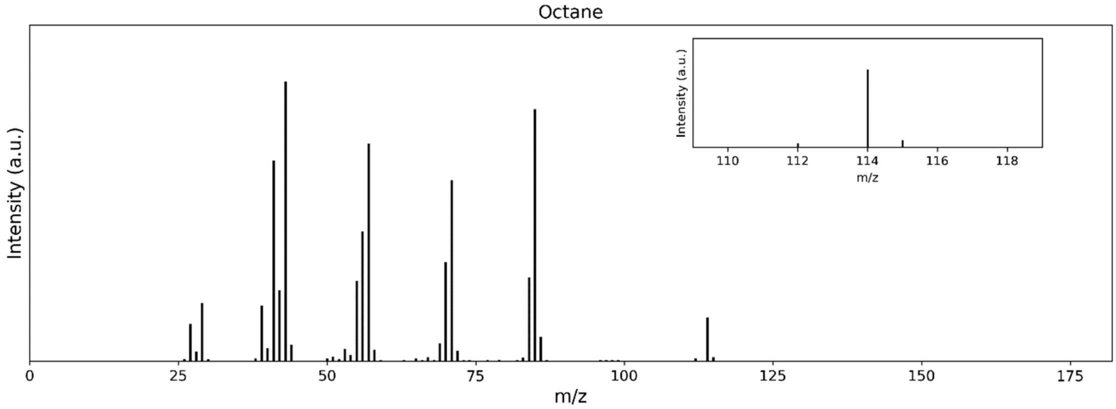
Octane
Intensity (a.u.)
m/z
0
25
50
75
100
125
150
175
110
112
114
116
118

\

Nonane

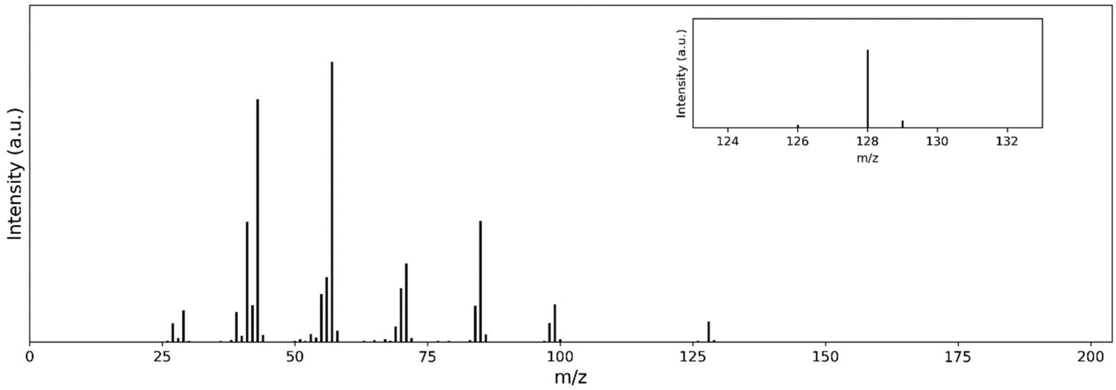

Decane

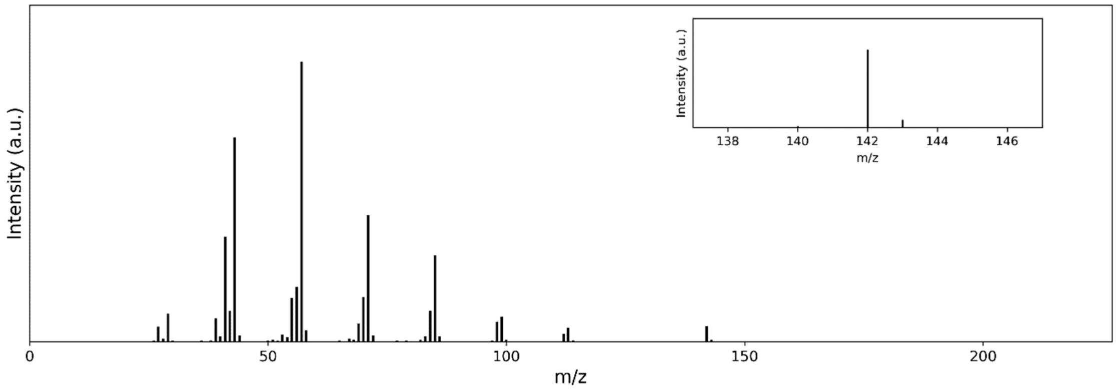

Undecane

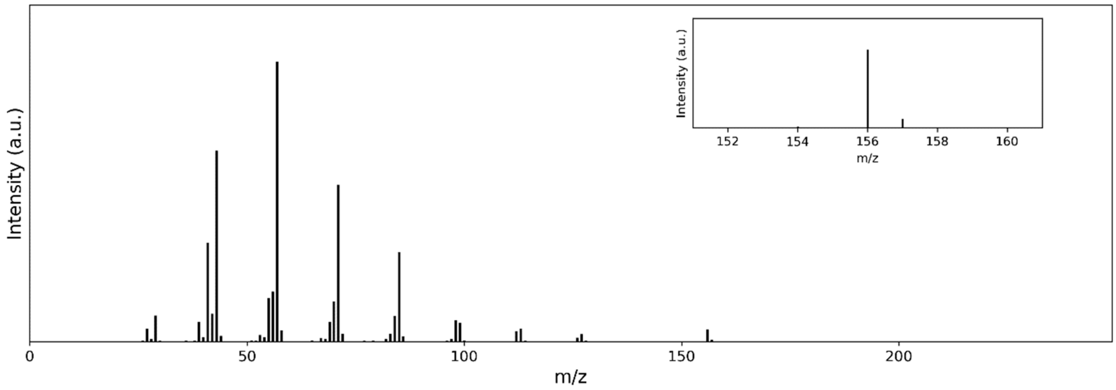

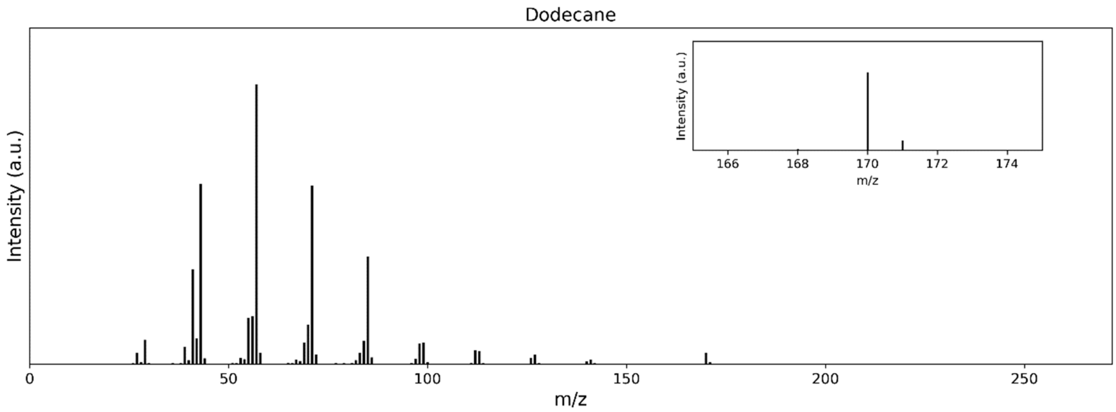
Dodecane
Intensity (a.u.)
m/z
0
50
100
150
200
250
166
168
170
172
174

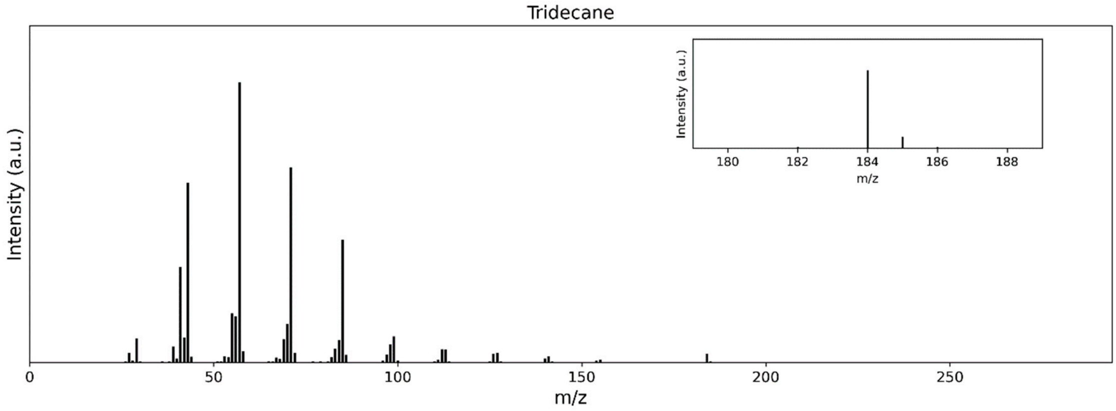
Tridecane
Intensity (a.u.)
m/z
0
50
100
150
200
250
180
182
184
186
188

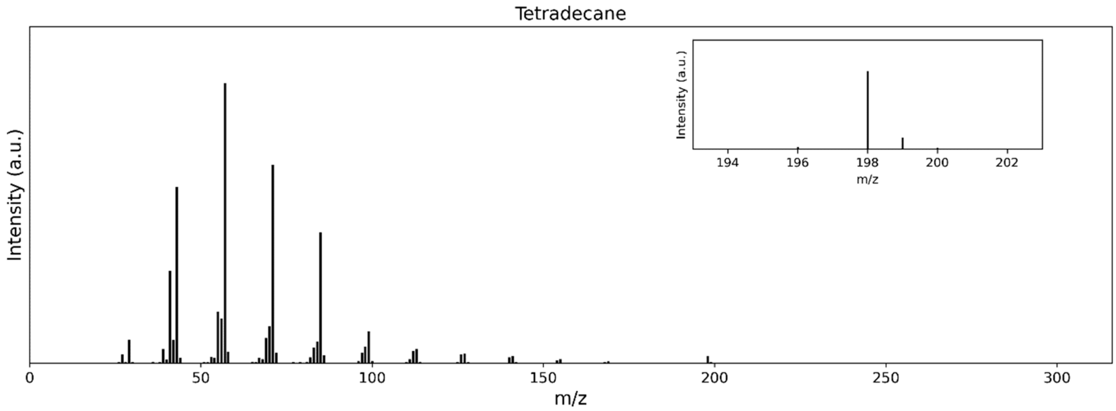
Tetradecane
Intensity (a.u.)
m/z
0
50
100
150
200
250
300
194
196
198
200
202

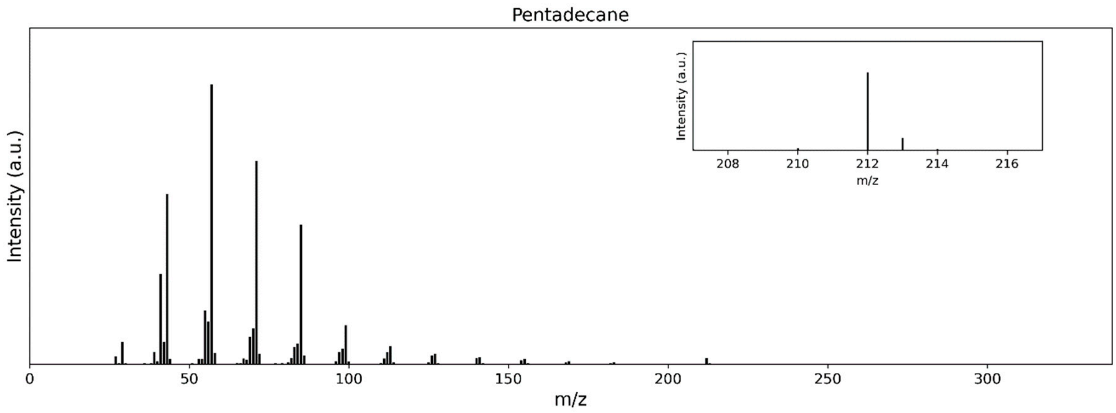
Pentadecane
Intensity (a.u.)
m/z
0
50
100
150
200
250
300
Intensity (a.u.)
m/z
208
210
212
214
216

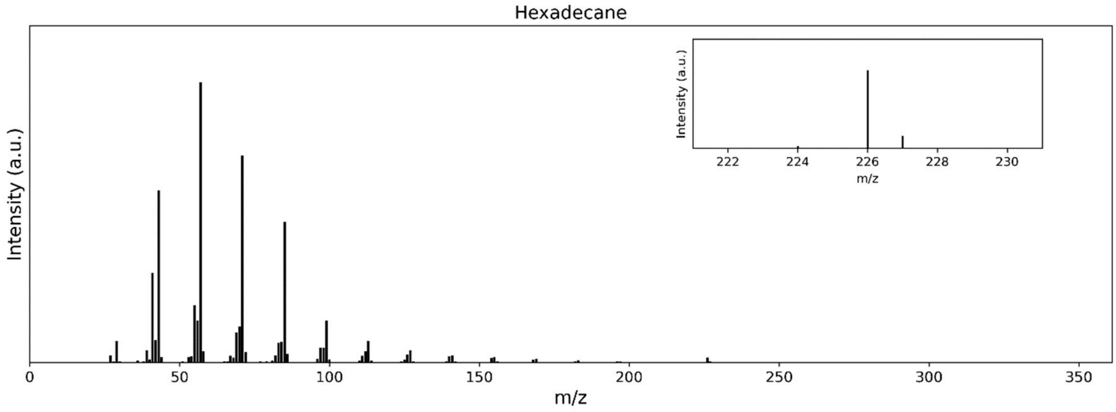
Hexadecane
Intensity (a.u.)
m/z
0
50
100
150
200
250
300
350
Intensity (a.u.)
m/z
222
224
226
228
230

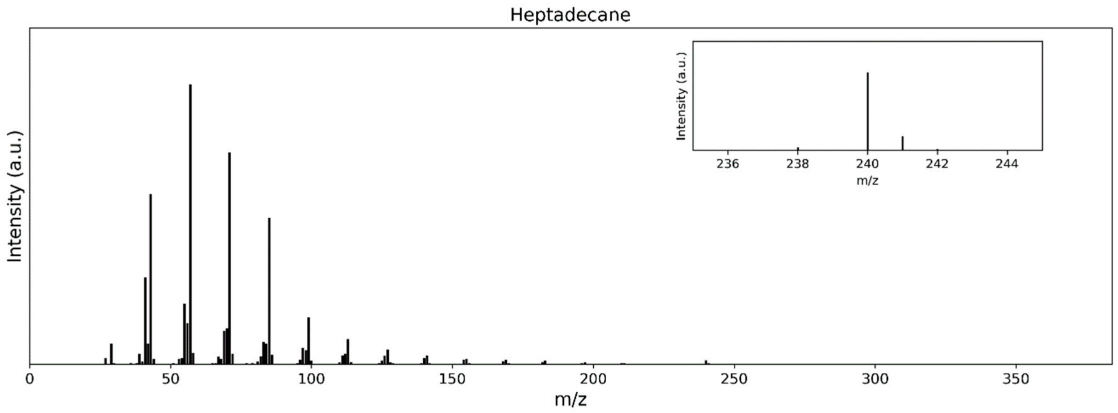
Heptadecane
Intensity (a.u.)
m/z
0
50
100
150
200
250
300
350
236
238
240
242
244

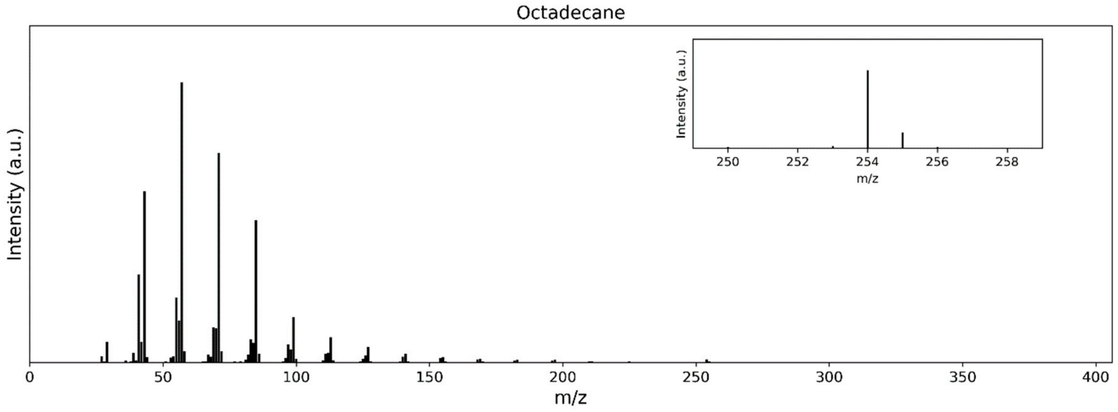
Octadecane
Intensity (a.u.)
m/z
0
50
100
150
200
250
300
350
400
250
252
254
256
258
